# Are we ready to collaborate? The interprofessional collaborative competencies of healthcare professionals in the Global South context

**DOI:** 10.3389/fmed.2022.904658

**Published:** 2022-10-21

**Authors:** Diantha Soemantri, Ardi Findyartini, Retno Asti Werdhani, Sukamto Koesnoe, Debie Dahlia

**Affiliations:** ^1^Department of Medical Education, Faculty of Medicine, Universitas Indonesia, Jakarta, Indonesia; ^2^Center for Administration, Health Sciences Cluster, Universitas Indonesia, Depok, Indonesia; ^3^Department of Community Medicine, Faculty of Medicine, Universitas Indonesia, Jakarta, Indonesia; ^4^Department of Internal Medicine, Faculty of Medicine, Universitas Indonesia—Dr. Cipto Mangunkusumo General Hospital, Jakarta, Indonesia; ^5^Department of Medical Surgical Nursing, Faculty of Nursing, Universitas Indonesia, Depok, Indonesia; ^6^Universitas Indonesia Hospital, Depok, Indonesia

**Keywords:** interprofessional competency, health professions, CICS29, validity, interprofessional education

## Abstract

**Background:**

Current evidence of interprofessional collaboration suggests the importance of measuring and identifying the current state of the health professions’ interprofessional competencies. Therefore, this study was aimed at measuring the interprofessional competencies of health professionals in the Global South context using the validated CICS29.

**Materials and methods:**

This was a cross-sectional study involving 300 healthcare professionals of a newly established teaching hospital. Prior to the measurement of interprofessional competencies, the 29-items CICS29, which has been translated into Indonesian language, was revalidated using a confirmatory factor analysis (CFA). The 29 items of CICS29 were grouped into six subscales and each item was measured using a 5-point Likert scale. Data on gender, age, type of profession, and the length of working experience was also collected to identify whether discernible differences between grouping variables exists.

**Results:**

Prior to measuring the interprofessional competencies, the validity of the instrument was established. Based on the CFA, the same six-factor model was found in the current study. The Indonesian CICS29 was reliable, with Cronbach alpha values of 0.921 for the whole instrument and that of each subscale ranged between 0.656 and 0.726. The mean total score of CICS29 was 128.53 (out of 145), ranged from 123 to 133.40 obtained by pharmacists and dentists respectively. No significant differences of CICS29 scores were found between grouping variables.

**Conclusion:**

The current study has revealed relatively good interprofessional competencies of healthcare professionals working in a newly established teaching hospital in the Global South healthcare context. Measuring the interprofessional competencies serves as baseline for further intervention to nurture and maintain collaborative practice. In addition, the current study has further proven the cross-cultural validity of CICS29, thus appropriate to be utilized in different setting and context.

## Introduction

The World Health Organization asserts the importance of interprofessional collaboration (IPC) practice to ensure safe and optimum patient care (1). IPC, as defined by Reeves et al. (2), is “the process by which different health and social care professional groups work together to positively impact care” (p. 7). Based on the systematic review published in the Cochrane Database of Systematic Reviews, Reeves et al. concluded that there are four types of IPC practice interventions: externally facilitated interprofessional activities, interprofessional rounds, interprofessional meetings, and interprofessional checklists (2). The systematic review suggested that these IPC practices could be effective in improving some clinical processes or outcomes, although the number of studies is small and there are limitations in terms of the studies’ methodologies. Another recent systematic review and meta-analysis of randomized clinical trials by Pascucci et al. identified some positive outcomes of IPC in management of chronic conditions, although only a few studies had a moderate level of evidence (3). Some of the clinical outcomes are duration of hospitalization, reduction of glycated hemoglobin level, and low-density lipoprotein level. The authors concluded that the positive outcomes resulted from more coordinated and patient-centered care and improved quality of care.

Despite the impact of interprofessional education (IPE) on patient care that has started to emerge, Reeves et al. argued that understanding of the collaboration process is still lacking, including how such collaboration affects clinical outcomes and processes (2). The authors further suggested that future research should focus on how collaboration is conceptualized and measured. Irajpour and Alavi identified power differentials as factors influencing the interactions among health workers (4). Despite the extensive efforts to design and implement interprofessional education program, understanding how each profession perceive their power and other professions’ power remains important (5). Mickan et al. in their case studies of IPE in several countries, both developed and developing, found common challenges in IPC which are the importance of good team functioning, a supportive system, including information management system and shared electronic health records and also clear protocols of case management (6). However, a study of IPC in a resource-limited setting showed how there are prominent professional hierarchy which inhibit collaboration, shortage of healthcare professionals and barriers of communication between healthcare professionals (7). Healthcare systems in a resource-limited setting are typically characterized by high patient load but limited resources, both human and infrastructures (8), in line with the classical characteristics of Global South context such as poverty, low health resources and limited access to medical education (9). Therefore, Nyoni et al. (7) suggested that there is a need for continued training on IPC in the healthcare setting and also to use the distributive leadership strategy to narrow the professional hierarchical gap.

Aside from understanding the nature of the collaborative practice, it is also important to learn about the interprofessional competencies of health care professionals. Measuring the interprofessional competencies of health care professionals is necessary given the role of health care professionals, especially those working in the academic health setting, as interprofessional role models for students (10). In a systematic review, Oates and Davidson identified nine instruments to measure the outcomes of IPE and collaborative practice in the health professions education setting and found a lack of evidence to support the instruments’ construct validity (11). This particular systematic review only accounts for the instruments used in the pre-qualification setting. However, an instrument called the Chiba Interprofessional Competency Scale (CICS29) was developed by Sakai et al. to measure the interprofessional competencies of health professionals (12). The 29-item scale was developed in a Japanese health care context through a series of instrument development steps. Its six subscales—namely attitudes and beliefs as a professional, team-management skills, actions for accomplishing team goals, providing care that respects patients, attitudes, and behaviors that improve team cohesion, and fulfilling one’s role as a profession—are considered compatible with the domains of IPC (13). The domains of IPC include roles and responsibilities, teams and teamwork, interprofessional communication, values, and ethics for interprofessional practice. These four domains are targeted toward delivering patient/family oriented or community/population-oriented healthcare services (13).

Several studies on measuring interprofessional competencies, either in pre-qualification or post-qualification settings, have been conducted in Indonesia. Three studies from Syahrizal et al., Dewi et al., and Lestari et al. measured health professions students’ readiness for and perceptions toward IPE (14–16), while Soemantri et al. translated the CICS29 into Indonesian language and provided evidence of its validity and used it in measuring the interprofessional competencies of health professions students (17). Based on a confirmatory factor analysis (CFA), they identified a good fit between the initial CICS29 model and the final one, following language adaptation. One study from Yusra et al. was conducted in the Indonesian healthcare professional setting in order to measure healthcare professionals’ perceptions toward IPC (18). The authors used Collaborative Practice Assessment Tool (CPAT) to assess levels of collaboration and identify strengths and weaknesses in collaborative practice (19). The final version of the Indonesian CPAT is slightly different from the original one with three items from the original CPAT discarded; indicating further investigation of the stability of the factors in the instrument in future research. Furthermore, because the CPAT consists of 56 items, the number of respondents in Yusra et al.’s study (*n* = 304) was considered relatively inadequate for a factor analysis (18).

Current evidence of IPC suggests the importance of measuring and identifying the current state of health professionals’ interprofessional competencies to make an informed decision about any intervention programs to improve hospital collaborative practice. Given the limited evidence available related to the healthcare professionals’ interprofessional competencies from the Global South healthcare context, with its typical characteristics such as limited healthcare resources, we argue for the need to measure it. The 29-item CICS29 is assumed to be the fit-for-purpose instrument since its validity has been established through CFA. Therefore, the aim of this study is to measure the interprofessional competencies of health professionals working in a hospital setting within the Global South context using the CICS29.

## Materials and methods

### Study context

The study was conducted at a university hospital which was recently established. One of the hospital’s missions is to conduct IPE, and the hospital also aims to provide interprofessional collaborative health care services.

### Study design

This single-site study employed a cross-sectional design to measure the interprofessional competencies of health professionals working at one university hospital using the Indonesian CICS29. Prior to analyzing the interprofessional competencies of healthcare professionals in this study, the validity of the instrument was previously established through calculating the internal consistency and conducting the CFA.

### Instrument

The CICS29 consists of 29 items grouped into six subscales (12). Each item is measured using a 5-point Likert scale, from five (“always”) to one (“never”), with a maximum possible total score of 145. Demographic data such as gender, age, type of profession, and years of working experience was obtained. The CICS29 has previously undergone forward and backward translation into Indonesian language in the study by Soemantri et al. (17).

### Data collection

Four hundred and fifty-seven healthcare professionals, including doctors, nurses, pharmacists, dentists, public health officers, and other allied health professionals, were invited to participate in the study. Three hundred health care professionals (65.6%) participated in this study. The details of the participants are provided in Table 1. Based on the requirement for factor analysis, which is 10 participants for each item in the instrument under study (20) and with the calculated minimum sample size of 209, the sample of health care professionals obtained in this study was deemed sufficient. The anonymous instrument was administered online using Google Forms, and the invitation was sent to potential participants through email and WhatsApp. The instrument was administered between October and December 2020. By completing the instrument, the participants provided their consent to participate in the study.

**TABLE 1:** Demographic characteristics of the respondents (*N* = 300).

Characteristics	*N*	%
**Age (years)**		
20–29	228	76
30–39	62	20.7
40–49	6	2
50 and above	4	1.3
**Gender**		
Male	59	19.7
Female	241	80.3
**Educational background**		
Vocational study	11	3.6
Undergraduate study	236	78.7
Postgraduate study	53	17.7
**Length of working experiences in the current hospital**		
1–15 months	186	62
16–30 months	103	34.3
Above 30 months	11	3.7
**Profession**		
Medicine	36	12.0
Dentistry	5	1.7
Public health	3	1.0
Nursing	244	81.3
Pharmacy	6	2.0
Other allied health professionals	6	2.0
**Working status**		
Part time	266	88.7
Full time	34	11.3

### Data analysis

To ensure the validity of the instrument, the data first underwent CFA using Stata 14 software to confirm the CICS29 model as compared with the original one. Following the confirmation of the CICS29 model, the internal consistency of the instrument was calculated. Further analysis was then conducted using SPSS 22.0 to examine the distribution of CICS29 scores and to identify whether discernible differences in the measurement results existed in relation to several variables such as age, gender, types of professions, educational background (vocational/undergraduate/postgraduate), working status (full time/part time), and length of working experience.

## Results

### The validity of the Indonesian CICS29

**FIGURE 1:**
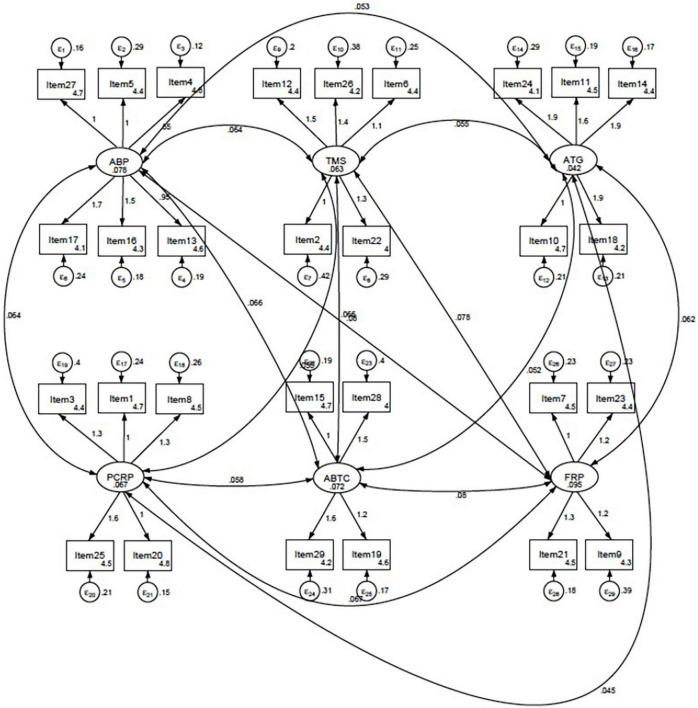
Final model of the CICS29 in the current study established through confirmatory factor analysis (CFA). ABP, attitudes and beliefs as a professional; TMS, team-management skills; ATG, actions for accomplishing team goals; PCRP, providing care that respect patients; ABTC, attitudes and behaviors that improve team cohesion; FRP, fulfilling one’s role as a professional.

The descriptive analysis was performed first to examine the validity of each CICS29 item by identifying the item-total correlation and Cronbach’s alpha of each item (Table 2). A CFA was then performed (Figure 1), which confirmed the previous models, not only the one by the original developer, Sakai et al. (12), but also that of Soemantri et al. (17) in their validation study of the Indonesian CICS29 in the medical and healthcare professions education setting. The subscales in the current Indonesian CICS29 have again proven their comparability to the original subscales in the model developed by Sakai et al.: (1) attitudes and beliefs as a professional, (2) team-management skills, (3) actions for accomplishing team goals, (4) providing care that respects patients, (5) attitudes and behaviors that improve team cohesion, and (6) fulfilling one’s role as a professional (12). Based on Hu and Bentler’s two-index presentation strategy (21), the combined value of the root mean square error of approximation (0.066) and standardized root mean square residual (0.057) indicated the goodness of fit of the current model.

**TABLE 2:** The Indonesian version of CICS29 items.

Subscales	Item numbers	Item-total correlation (r)	Cronbach’s alpha if item deleted	Items
Attitudes and beliefs as a professional (ABP)	27	0.533	0.918	*Saya selalu berusaha memperbaiki keterampilan saya* (I constantly strive to improve my performance)
	5	0.444	0.919	*Saya selalu melakukan refleksi terhadap tata laksana yang saya lakukan* (I always reflect on the care that I have provided)
	4	0.385	0.920	*Saya berusaha menjadi sosok profesional* (I strive to be a professional)
	17	0.617	0.917	*Saya dapat melakukan tata laksana pasien berdasarkan bukti terkini* (I practice evidence-based care)
	16	0.640	0.916	*Saya dapat menjelaskan dasar keilmuan tata laksana yang saya lakukan* (I am able to explain the basis for care to anyone)
	13	0.499	0.918	*Saya melakukan pekerjaan sesuai keilmuan yang diajarkan* (I am able to apply updated expert knowledge to actual practice)
Team-management skills (TMS)	12	0.608	0.917	*Saya memahami ruang lingkup dan batasan kerja anggota tim* (I understand the scope and limits of my team members’ work)
	26	0.476	0.919	*Saya mempertimbangkan kesibukan dan kecepatan kerja anggota tim lain* (I respect my team members’ busy schedules and work pace)
	6	0.474	0.919	*Saat terjadi masalah, saya dapat bekerja sama dengan anggota tim lain untuk memecahkannya* (I cooperate with my team members to try to solve problems when the team is not functioning well)
	2	0.376	0.921	*Saat terjadi konflik antar anggota tim, saya berusaha menyesuaikan diri untuk menyelesaikan konflik tersebut* (I reconcile conflicts among team members)
	22	0.473	0.919	*Saya mengetahui pada kondisi apa masalah mudah terjadi* (I know when problems within the team are likely to arise)
Actions for accomplishing team goals (ATG)	24	0.549	0.918	*Saya dapat menjelaskan pencapaian tim* (I am able to explain the results of my team’s initiatives)
	11	0.566	0.918	*Saya dapat menyesuaikan perilaku untuk mencapai tujuan tim* (I am able to adjust my practices to achieve the team’s objectives)
	14	0.662	0.916	*Saya dapat menyesuaikan pendapat selaras dengan tujuan tim* (I am able to coordinate the opinions of myself and my team members in light of the team’s objective)
	10	0.403	0.920	*Saya mendukung pengembangan kompetensi masing-masing profesi* (I provide necessary support to my team members depending on their professional competencies)
	18	0.633	0.916	*Saya dapat melakukan evaluasi kerja tim secara objektif* (I am able to objectively evaluate whether the team is operating well)
Providing care that respects patients (PCRP)	3	0.414	0.920	*Saya tidak hanya menghormati kepentingan pasien, tetapi juga memperhatikan keinginan keluarga pasien* (I respect not only the wishes of the patient but also those of the patient’s family)
	1	0.395	0.920	*Tata laksana pasien dilakukan dengan memperhatikan otonomi pasien* (I keep patient independence in mind when providing care)
	8	0.513	0.918	*Saya melibatkan pasien dalam proses pengobatan* (I interact with patients to help them make their own decisions)
	25	0.569	0.917	*Dalam interaksi dengan pasien, saya menyesuaikan dengan karakteristik dan kondisi pasien* (I change my manner of interacting with patients based on their characteristics and situations)
	20	0.498	0.919	*Saya selalu berusaha memberikan tata laksana terbaik untuk pasien* (I seek the best way to care for patients)
Attitudes and behaviors that improve team cohesion (ABTC)	15	0.480	0.919	*Saya berusaha berkomunikasi dengan cara terbaik dengan anggota tim dari profesi lain* (I consciously create opportunities for communication with other professionals)
	28	0.487	0.919	*Saya secara rutin membahas tata laksana pasien dengan anggota tim dari profesi lain* (I discuss ideal patient care with other professionals daily)
	29	0.569	0.917	*Dalam pertemuan, saya berusaha menciptakan suasana yang memudahkan tukar pikiran dengan anggota tim profesi lain* (I try to create a suitable atmosphere during meetings wherein it is easy for other professionals to speak)
	19	0.565	0.918	*Saya berusaha membangun hubungan baik dalam melakukan pekerjaan dalam tim interprofesi* (I strive daily to create good interpersonal relationships between professionals)
Fulfilling one’s role as a professional (FRP)	7	0.520	0.918	*Saya dapat menerima masukan sesuai kepakaran profesi lain* (I am able to express opinions in front of other professionals based on my expert knowledge)
	23	0.571	0.917	*Saya dapat menjalankan peran profesi sesuai kebutuhan tim* (I fulfill my professional role as required by my team)
	21	0.626	0.917	*Saya memahami lingkup pengetahuan dan keterampilan sesuai profesi* (I understand the scope of what can be accomplished through professional expertise and skills)
	9	0.476	0.919	*Saat terjadi konflik antar profesi, saya akan memberikan pendapat sesuai keilmuan saya* (I am able to state my opinions when necessary from the viewpoint of my professional expertise, even if doing so creates friction with other professionals)

Sentences written in italics are in Indonesian (the English versions of the sentences are provided in brackets).

The validity of the instrument was further established by determining the reliability of the instrument as a whole and of each subscale. The Cronbach alpha value of the whole instrument was 0.921, and the value for each of the subscales was as follows: attitudes and beliefs as a professional (ABP), 0.732; team-management skills (TMS), 0.621; actions for accomplishing team goals (ATG), 0.726; providing care that respects patients (PCRP), 0.669; attitudes and behaviors that improve team cohesion (ABTC), 0.657; and fulfilling one’s role as a professional (FRP), 0.656.

### Interprofessional competency of health care professionals

Using the validated CICS29, we examined the interprofessional competencies of the participants. The mean total CICS29 score was 128.53 (out of 145, 88.6%), ranging from 123 (mean CICS29 score of pharmacist profession) to 133.40 (mean CICS29 score of dentist profession). Based on a one-way ANOVA, we found that there were no significant differences between the total mean score of each profession group, *F*_(5,294)_ = 0.644, *p* = 0.666. The scores of each subscale also did not significantly differ between profession groups, ABP, *F*_(5,294)_ = 1.470, *p* = 0.200; TMS, *F*_(5,294)_ = 1.147, *p* = 0.336; ATG, *F*_(5,294)_ = 0.239, *p* = 0.945; PCRP, *F*_(5,294)_ = 1.751, *p* = 0.123; ABTC, *F*_(5,294)_ = 1.624, *p* = 0.154; and FRP, *F*_(5,294)_ = 1.048, *p* = 0.389. Complete results of the mean scores of the total CICS29 and its subscales are presented in Table 3. We acknowledged that our results must be interpreted with caution given the large differences in sample size in certain groups; however, most of the homogeneity of variance (Levene’s) tests showed non-significant results, which inferred that equal variances can be assumed. Significant Levene’s test results only occurred in the first subscale (ABP).

**TABLE 3:** Mean scores of total CICS29 and each of the subscales.

Profession	*N*	Mean score						
		
		Total CICS29	Subscale 1: ABP	Subscale 2: TMS	Subscale 3: ATG	Subscale 4: PCRP	Subscale 5: ABTC	Subscale 6: FRP
Doctors	36	128.53	27.61	20.89	21.75	23.11	17.11	18.06
Dentists	5	133.40	27.80	22.00	22.40	24.20	18.20	18.80
Nurses	244	128.55	26.99	21.48	21.93	22.92	17.64	17.59
Pharmacists	6	123.00	25.50	20.67	21.33	21	16.17	18.33
Public health officers	3	129.67	28.33	21.33	22.33	23.33	16.67	17.67
Other allied health professionals	6	129.00	27.17	22.50	22	22.50	17.17	17.67
*P*-value from one-way ANOVA		0.666	0.200	0.336	0.945	0.123	0.154	0.389

The analysis also included the differences in the mean total CICS29 scores based on the grouping variables using the appropriate statistical analysis. The independent *t*-test analysis demonstrated no significant correlations on gender, *t*_(298)_ = 1.881, *p* = 0.061, and working status, *t*_(298)_ = 0.582, *p* = 0.561. Similar results were found using the one-way ANOVA on the variable of participants’ educational background, *F*_(3,296)_ = 0.908, *p* = 0.437. A significant regression equation was not found between mean total CICS29 scores with age and length of working experiences, *F*_(3,296)_ = 0.534, *p* = 0.660, with an *R*^2^ of 0.005. The predicted mean CICS29 total score is equal to 127.506–0.040 (length of working experiences) + 0.146 (age).

## Discussion

The findings of the CFA have demonstrated the validity of the CICS29 as an instrument to measure health professionals’ interprofessional competencies. The original CICS29 has also been validated in the Italian setting (22), where the authors confirmed the six-factor model as originally developed by Sakai et al. (12), based on the data obtained from 530 healthcare professionals. The current study in which the Indonesian version of CICS29 was administered to healthcare professionals in a single hospital demonstrated the same six-factor model (ABP, TMS, ATG, PCRP, ABTC, and FRP). Furthermore, the Cronbach alpha values are comparable to those of other studies. For example, for the ATG subscale, the Cronbach alpha value in the current study was 0.726, whereas Tonarelli et al. obtained a value of 0.77 for the same subscale (22).

Since the CICS29 has undergone several CFAs in different settings and countries and still retains its original six-factor model, we argue for the strength and quality of the instrument, which further support the suitability of its use in measuring interprofessional competencies in the current setting. Peltonen et al. (23) in their scoping review identified 29 instruments measuring IPC and found few studies which have reported the construct validity of those instruments. Moreover, the authors also revealed that most studies included in their scoping review involved only two major groups of health professions (i.e., doctors and nurses). Therefore, we argue that our study has also supported the validity of CICS29 across professions since we involved six professional groups as study participants. Other studies utilizing the CICS29 have also involved more than two professions, for example psychologists, social workers, and radiology technicians (22), as well as pharmacists, dieticians, and rehabilitation-related therapists (12).

Using the valid Indonesian version of the CICS29, the interprofessional competencies of health professionals in this study were measured. Despite the unavailability of clear guidelines on how to categorize and interpret the CICS29 scores, the interprofessional competencies of the study participants are considered satisfactory because the mean total score of the CICS29 was around 88.6% of the maximum possible score. Other studies in the healthcare professional setting measured the perceptions of health care professionals toward IPC practice. For example, Soemantri et al. (24) conducted a study in another newly established teaching hospital using the Indonesian CPAT and found a median score of 205 (out of 265, 77.4%), whereas in one of the oldest teaching hospitals in the country, with the same instrument, Yusra et al. (18) obtained a median score of 205.5 (out of 265, 77.5%). Both studies were conducted in the Indonesian healthcare service setting; therefore, although direct comparison with the results of the current study cannot be made given the different instruments used, we can conclude that the perceptions toward collaborative practice and interprofessional competencies of health care professionals in Indonesia are relatively good.

Based on the breakdown of subscales, the relatively high CICS29 subscale scores indicated that most participants in this study have what it takes to become effective interprofessional team members. Since the CICS29 subscales represent the essential abilities and attitudes for effective IPC, summarized by Reeves (25) in his editorial review of various interprofessional competency frameworks. The ABP subscale indicates values and identity as a professional. Majima et al. (26) found that nurses in their study valued their work highly and this has led to increase job satisfaction. The TMS, ATG, ABTC, and FRP subscales relate to teamwork. Reeves (25) highlighted that teamwork involves clear roles and responsibility among team members, shared goals and responsibility, shared identity as a team and interdependence between members. The last subscale is PCRP which indicates the ability of healthcare professionals to provide patient-centered care. Dahlke et al. (27) demonstrated that older people and their families appreciate the delivery of healthcare services which have taken into account the characteristics of the elderly population. The data of total CICS29 and its subscales scores can serve as baseline data which can be re-evaluated following a particular intervention to improve collaborative practice, for example a study by Shikino et al. (28) has found increases in CICS29 scores after a simulation-based training for delirium management.

The study findings also demonstrate that there are no statistically significant differences in the CICS29 scores based on professions and other discerning variables such as age, gender, working status, and length of working experiences. Older age and longer working experiences have been found to be the factors that influence IPC (15). Because the hospital in which the study was conducted is a newly established hospital, most of the hospital’s health care professionals have similar characteristics, for example in terms of length of working experiences and age. These characteristics might be partly responsible for the attainment of a relatively similar level of interprofessional competencies. It is also likely that the hospital’s mission to provide collaborative healthcare services has imbued each individual to conduct collaborative practice. Soemantri et al. (24) found similar results in their study involving health care professionals in a newly established hospital and argued that power distance is narrow between health care professionals in that particular setting, which results in them having relatively similar perceptions toward collaborative practice. However, a study in an older hospital also found relatively positive attitudes and perceptions toward interprofessional collaboration (18) thus other factors play important roles in affecting interprofessional competencies. Dahlke et al. (29) summarized the four factors influencing collaboration process which are relational (professional power, hierarchy and socialization process), processual (time and space for collaboration), organization (system and resources to collaborate) and contextual issues (sociocultural, political and economic). Given these complex interconnected issues, the causes of positive perceptions toward IPC and good interprofessional competencies are very much multifactorial, for example when healthcare professionals in a certain hospital are very diverse, there needs to be stronger system and resources in place to facilitate the IPC.

Based on the study findings, several implications can be outlined. First, measuring healthcare professionals’ interprofessional competencies is important to serve as baseline for assessing the effectiveness of any interventions to improve collaborative practice. Second, the CICS29 is proven to be one of the instruments with cross-cultural validity, thus using the same instrument results from different countries can be directly compared to further inform IPC practice throughout the world, including within the Global South healthcare context. This is perhaps even more important for the Global South healthcare context since the hierarchical professional boundaries can be more prominent (7). Third, based on the subscales of CICS29, each component of collaborative practice, starting from the teamwork skills, professional roles to individual professional’s identity can be assessed and intervened.

We acknowledge the limitation of the study in that it only involved one hospital in Indonesia. Therefore, the study might not be directly generalizable. As with other self-administered scales, there is a possibility for participants to provide socially desirable responses, which might not reflect the real situation. Despite these limitations, we believe that our study has contributed to the understanding of healthcare professionals’ interprofessional competencies in a Global South healthcare context. Measuring health care professionals’ interprofessional competencies could serve as the basis for intervention programs to further improve interprofessional competencies and enable more patient-centered and collaborative care. Our study has also established the construct validity of the Indonesian version of the CICS29, including cross-cultural validity. Further study is necessary to include more study sites and explore each of the factors which influence collaborative practice in more depth, which can lead to an understanding of how IPC can be further nurtured and maintained, especially in the Global South healthcare context. Moreover, a study to examine the relationships between interprofessional competencies and certain healthcare outcomes is also worthwhile.

## Conclusion

Measuring the interprofessional competencies of healthcare professionals in this study, using the CICS29 which has been proven to have cross-cultural validity, has advanced our understanding in terms of how they perceive their competencies, especially in a newly established hospital in the Global South healthcare context. The current study has identified relatively good interprofessional competencies and moreover, no differences in the competencies based on professions, age, and length of working experiences were found. Thus, an effective collaborative practice is to be expected, along with continued awareness that collaborative practice is indeed a complex construct that requires further in-depth exploration and observation.

## Data availability statement

The raw data supporting the conclusions of this article will be made available by the authors, without undue reservation.

## Ethics statement

The studies involving human participants were reviewed and approved by Research Ethics Committee, Faculty of Medicine, Universitas Indonesia. The patients/participants provided their written informed consent to participate in this study.

## Author contributions

DS led the study from the concept development, data collection, data analysis, and manuscript development and wrote the first draft of the manuscript. AF, RW, SK, and DD contributed to data collection, analysis, reviewed, edited, and finalized the manuscript. All authors have read and approved the final version of the manuscript.
